# Two independent LAMP assays for rapid identification of the serpentine leafminer, *Liriomyza huidobrensis* (Blanchard, 1926) (Diptera: Agromyzidae) in Australia

**DOI:** 10.1038/s41598-023-49472-9

**Published:** 2023-12-15

**Authors:** Xiaocheng Zhu, David Gopurenko, Joanne C. Holloway, John D. Duff, Mallik B. Malipatil

**Affiliations:** 1 NSW Department of Primary Industries, Wagga Wagga Agricultural Institute, Wagga Wagga, NSW 2650 Australia; 2 Queensland Department of Agriculture and Fisheries, Warrego Highway, Gatton, QLD 4343 Australia; 3 Agriculture Victoria Research and La Trobe University, 5 Ring Road, Bundoora, VIC 3083 Australia

**Keywords:** Agricultural genetics, Taxonomy, DNA sequencing

## Abstract

*Liriomyza huidobrensis* is a leafminer fly and significant horticultural pest. It is a quarantine listed species in many countries and is now present as an established pest in Australia. *Liriomyza huidobrensis* uses a broad range of host plants and has potential for spread into various horticultural systems and regions of Australia. Rapid in-field identification of the pest is critically needed to assist efforts to manage this pest. Morphological identification of the pest is effectively limited to specialist examinations of adult males. Generally, molecular methods such as qPCR and DNA barcoding for identification of *Liriomyza* species require costly laboratory-based hardware. Herein, we developed two independent and rapid LAMP assays targeted to independently inherited mitochondrial and nuclear genes. Both assays are highly sensitive and specific to *L. huidobrensis*. Positive signals can be detected within 10 min on laboratory and portable real-time amplification fluorometers. Further, we adapted these assays for use with colorimetric master mixes, to allow fluorometer free in-field diagnostics of *L. huidobrensis*. Our LAMP assays can be used for stand-alone testing of query specimens and are likely to be essential tools used for rapid identification and monitoring of *L. huidobrensis*.

## Introduction

Larvae of leafminer insects develop in, and feed on, parenchyma tissues between leaf surfaces of host plants, leaving behind distinctive mined tunnels and frass deposits. Leafminers can adversely affect host plant health by reducing leaf photosynthesis, increasing leaf decay, and allowing entry of diseases into hosts^1^. Leaf mining behaviours have evolved in four insect orders and are present in nine phytophagous fly families. They are prevalent among most of the around 3163 species of Agromyzidae Fallén, 1823 that collectively feed off over 140 families of host plants^2–4^. Various leafminer Agromyzids are agricultural pests with some being highly polyphagous across economically important host plants and are therefore significant pests of quarantine importance to international trade. *Liriomyza huidobrensis* (Blanchard, 1926), *L. sativae* Blanchard, 1938 and *L. trifolii* (Burgess, 1880) are prevalent among these significant pests.

These three leafminer species evolved in the Americas, but are naturalised pests in most other continents, including Australia where each has recently established in different regions^5–7^. They are collectively ranked as number 20 in the current Australian National Priority Plant Pests list^8^. Each pest is recognised as a significant risk to the production of a variety of economically important horticultural crops and ornamental plants. In particular, *L. huidobrensis*, commonly known as serpentine leafminer (referred herein as SLM), was identified during 2020 surveillance in the greater Sydney region of NSW as a novel invasive pest. SLM causes extensive foliar damage to commercial vegetable crops grown in the region including beans, cucumbers and Asian leafy greens^7^.

SLM can affect a broad variety of agricultural, ornamental and weed host plants in Australia^7^, many of which are also hosts used by the two other introduced *Liriomyza* pests in Australia^9^. The likelihood of spread of these *Liriomyza* pests into diverse agricultural and ecological systems and regions in Australia is high^9^. Subsequently affected agricultural and ornamental industries will need to develop tailored integrative pest management strategies to deal with each pest according to their biology and interactions with hosts and other leafminer species^10–13^. In this context, correct and rapid species identification of SLM under field conditions is critical for timely control and management of outbreaks, particularly if SLM disperses into new areas or onto novel host plants.

There are 18 naturally present *Liriomyza* species in Australia^14^. Endemic leafminer species are often not pests of agricultural concern. Cited occurrences of most of these species are scarce or only historically reported (refer Atlas of Living Australia; https://www.ala.org.au/). Direct identification of leafminer species in the field is difficult and subject to observer error. Readily observable leaf mines on host plants flags the presence of pest leafminer activity. In a few cases, the mine patterns and host identity may be indicative of a particular pest species^7,15,16^. In-field visual identification of adult Agromyzid leafminer species is not considered practical due to their small size (Agromyzids range in size from 0.9 to 5.6 mm) and the subtlety of morphological features used in their diagnostics. Many Agromyzid species lack a formal description, and most of the described species can only be distinguished from siblings by a few observable morphological characters. Furthermore, female adult and early instar Agromyzids generally lack species-specific features, and most species identifications are reliant on dissection of male adults and microscopic examination of their genitalia. Typically, during pest leafminer surveillance, species identifications require laboratory based taxonomic examinations of male adult flies either captured directly on hosts or raised from instars in leaf mines sampled from affected host plants. Development from egg to assayable adult in these latter instances can take 15–30 days^17,18^, and this can delay an alert to the presence of a priority pest and subsequent management responses.

Alternatively, molecular genetic methods can provide species level identifications of leafminer flies and key Agromyzid pests. The maternally inherited mitochondrial cytochrome *c* oxidase subunit I (COI) gene has featured prominently as a targeted locus for molecular identification of some economically important Agromyzid species^19–21^. Nucleotide sequences of the 5’ COI DNA barcode region^22^ linked to vouchered specimens are reported for genetic identification of important leafminer pest species^19,23^. These sequence references have formed the basis for further development of laboratory and or point of need genetic diagnostic methods to identify invasive *Liriomyza* pests in Australia^24,25^ and elsewhere^20,26,27^. Sequences of nuclear encoded genes, including 28S and carbamoyl-phosphate synthetase 2 (CAD), reported for phylogenetic analyses of some Agromyzids^3^ and *Liriomyza*^28^ offer additional advantages for species identifications of pest leafminers. Comparative sequence analysis of independently inherited mitochondrial and nuclear loci were used to identify morphologically cryptic *Liriomyza* species^28^, and to test the direction of interspecific hybridisation between closely related *Liriomyza* species^29^. Recently, a quantitative Polymerase Chain Reaction (qPCR)-based molecular identification method was developed for *L. huidobrensis*^27^, addressing a critical need for *Liriomyza* biosecurity. However, this qPCR method has only been tested on a limited number of non-targeted species and still requires validation to confirm its applicability in Australia.

Genetic methods used for pest species identifications, such as qPCR and nucleotide sequencing, can take hours to days of laboratory processing time. This delay, coupled with delivery and registration of specimens at laboratories, increases the time required to provide an accurate identification and substantiated alert to the presence of a pest. Rapid in-field genetic diagnostics is preferable for a quick test confirmation of suspected SLM intercepts, but currently such systems are at primary stages of development or require substantive and or costly hardware.

Loop-mediated Isothermal Amplification (LAMP) is a low-cost technique for confirmation and or detection of target organisms^30^. LAMP incorporates a suite of oligo-primers specifically matched to the DNA of a target organism, and is designed to rapidly amplify linked copies of the target DNA. LAMP is well suited for in-field species identification of targeted pest insects, as it can test crude DNA lysates run on low-cost equipment for simple visual signalling of positive and negative test results^31^.

Here we report novel development and validation of sensitive LAMP assays for rapid and specific identification of SLM, against a selection of leafminer species sampled during recent surveys for SLM in Australia. Also, we report modifications of the assays to allow simplified in-field colorimetric visualisation of SLM LAMP test results.

## Results

### Assay design and optimization

**Figure 1:**
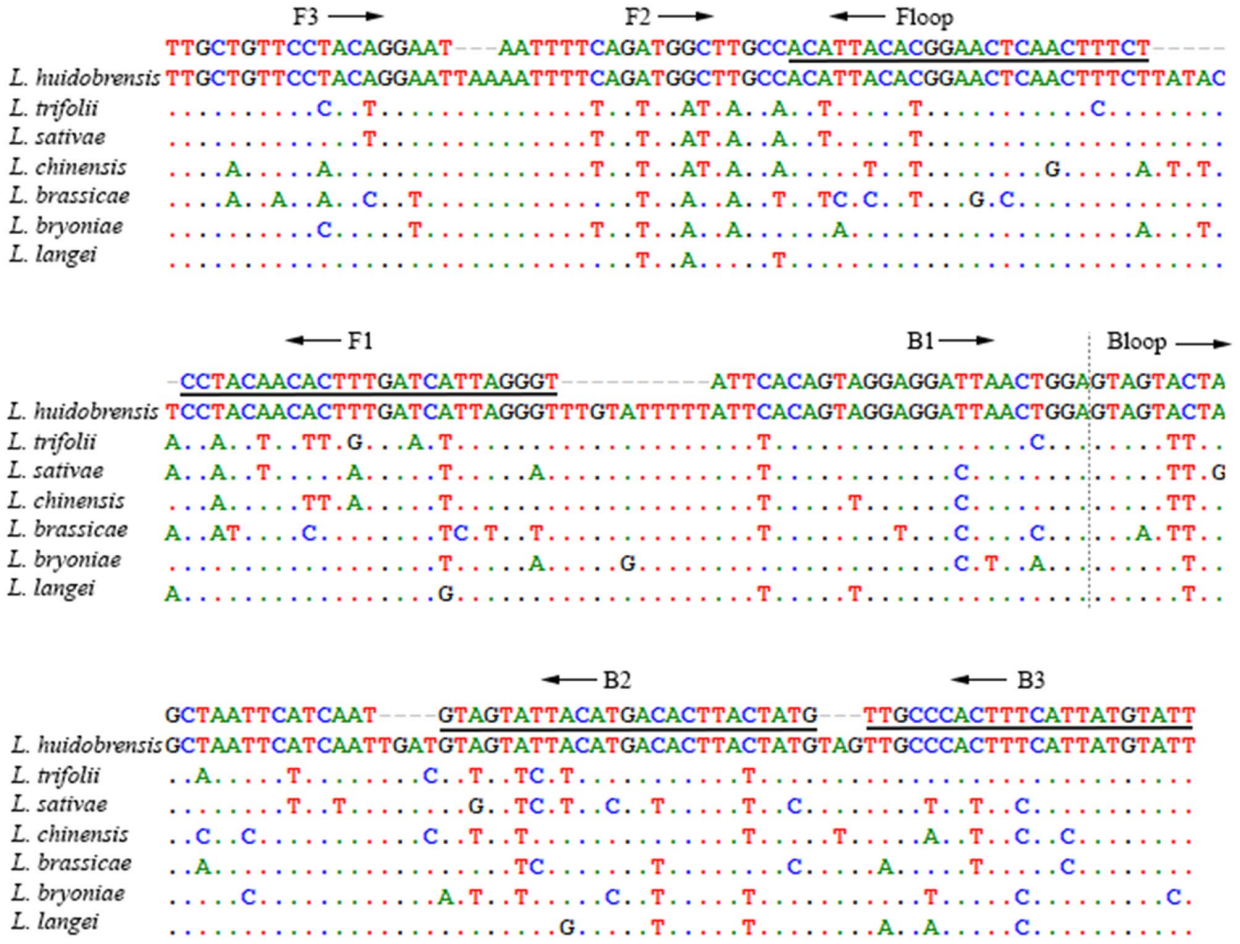
COI2377 LAMP primer anneal sites in partial COI alignment of *Liriomyza huidobrensis* and six other species of leafminer. Reverse primer sites are underlined. Arrows indicate the extension directions. Dotted line separate adjunct primer sites.

**Figure 2:**
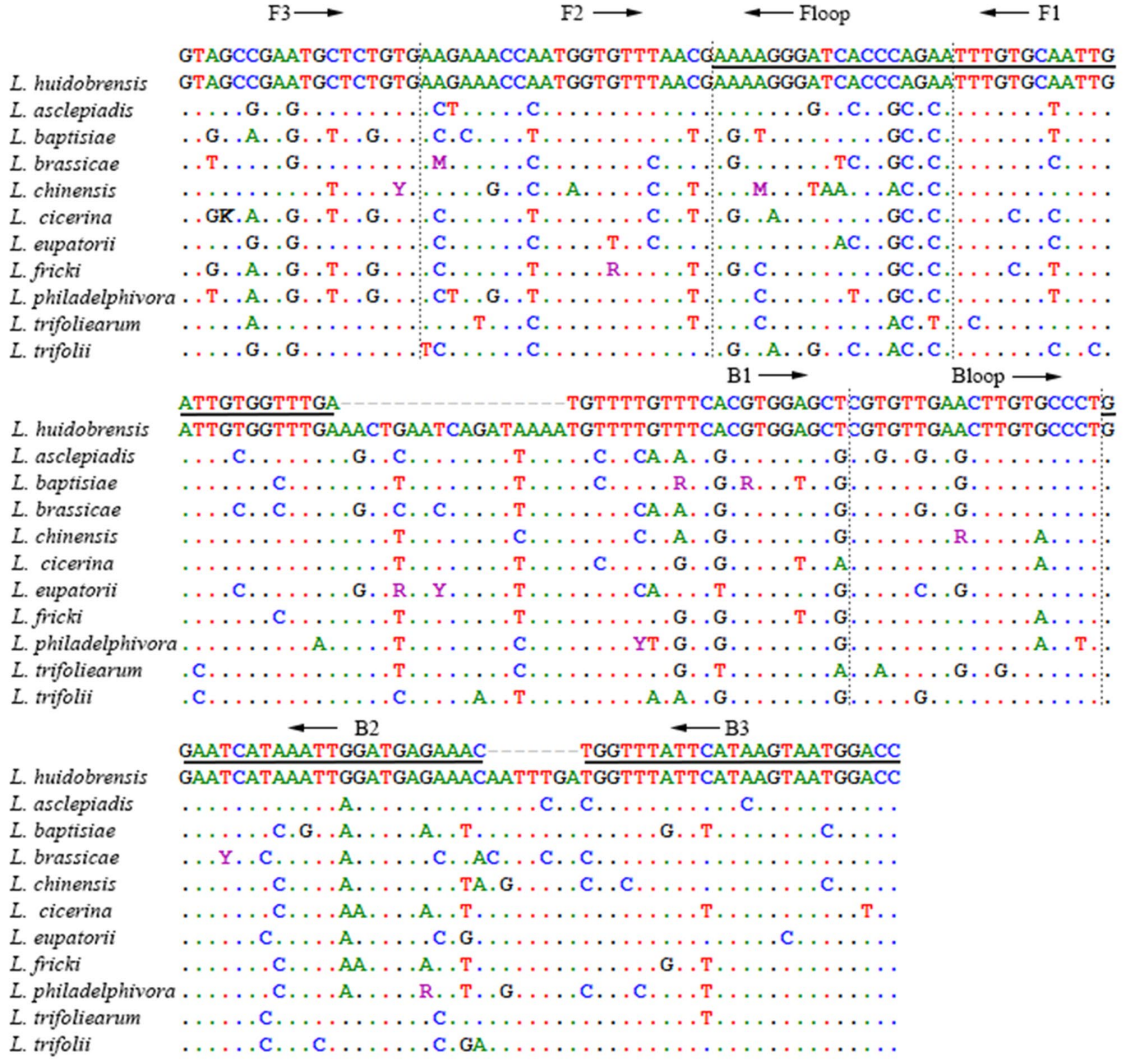
CAD263 LAMP primer anneal sites in partial CAD alignment of *Liriomyza huidobrensis* and ten other species of leafminer. Reverse primer sites are underlined. Arrows indicate the extension directions. Dotted lines separate adjunct primer sites.

We designed novel LAMP primers (Table 1) for the mitochondrial COI region and nuclear CAD region for *L. huidobrensis*. Both regions are highly variable among species (Figs. 1 and 2), with sufficient resolution to distinguish SLM from all sequenced species. The COI LAMP primer set (COI2377) targeted a 208 bp region downstream from the standard 5’ DNA barcode region, the CAD primer set (CAD263) targeted a 194 bp region. Some primers in sets were modified with Locked Nucleic Acids (Table 1) to increase the melting temperature.

**Table 1:** Details of LAMP primers and gBlock synthetic gene fragments designed in our study

Primer name	Sequence (5′-3′)	Gene	Target sequence
SLM_COI2377_F3	TTGCTGTTCCTAC[+A]GG[+A]AT	COI	208 bp
SLM_COI2377_B3	AATACATAATGAAAGTGGGCAA	COI	
SLM_COI2377_FIP	ACCCTAATGATCAAAGTGTTGTAGG**AATTTTCAGATGGCTTGCC**	COI	
SLM_COI2377_BIP	ATTCACAGTAGGAGGATTAACTGGA**CATAGTAAGTGTCATGTAATACT[+A]C**	COI	
SLM_COI2377_LF	[+A]GAAAGTTGAGTTCCGTGTAATGT	COI	
SLM_COI2377_LB	GTAGT[+A][+C]TAGCTAATTCATCAAT	COI	
COI gBlock	TTGCTGTTCCTACAGGAATggggTAAAATTTTCAGATGGCTTGCCggggACATTACACGGAACTCAACTTTCTTggggATACTCCTACAACACTTTGATCATTAGGGTTggggTGTATTTTTATTCACAGTAGGAGGATTAACTGGAgggGTAGTACTAGCTAATTCATCAATggggTGATGTAGTATTACATGACACTTACTATGgggTAGTTGCCCACTTTCATTATGTATT		
SLM_CAD263_F3	GTAGCCGAATGCTCTGTG	CAD	194 bp
SLM_CAD263_B3	GGTCCATTACTTATGAATAAACCA	CAD	
SLM_CAD263_FIP	TCAAACCACAATCAATTGCACAAA**AAGAAACCAATGGTGTTTAACG**	CAD	
SLM_CAD263_BIP	TGTTTTGTTTCACGTGGAGCT**GTTTCTCATCCAATTTATGATTCC**	CAD	
SLM_CAD263_LF	T[+T]C[+T][+G]GG[+T]GATCCCTTTT	CAD	
SLM_CAD263_LB	CGTGTTGAACTTGTGCCCT	CAD	
CAD gBlock	GTAGCCGAATGCTCTGTGcccAAGAAACCAATGGTGTTTAACGcccAAAAGGGATCACCCAGAATTTGTGCAATTGATTGTGGTTTGAAcccACTGAATCAGATAAAATGTTTTGTTTCACGTGGAGCTcccCGTGTTGAACTTGTGCCCTcccGGAATCATAAATTGGATGAGAAAcccCAATTTGATGGTTTATTCATAAGTAATGGACC		

F2 and B2 primer regions of FIP and BIP are bold. Locked Nucleic Acids were marked with [+]. Lower case bases in the gBlock sequences are our modifications to increase the annealing temperature.

For both assays, primer ratios (F3/B3: FIP/BIP: Floop/Bloop) were optimized at 1:6:3, with the final concentrations of 0.4, 2.4 and 1.2 µM, respectively. LAMP assays run using two different commercial isothermal master mixes (DR001 & DR004) were similar in duration to peak product amplification. However, the fluorescent intensity of assays was much higher when using master mix DR001. Subsequently, we used DR001 in all downstream LAMP assays.

### Assay sensitivities

**Figure 3:**
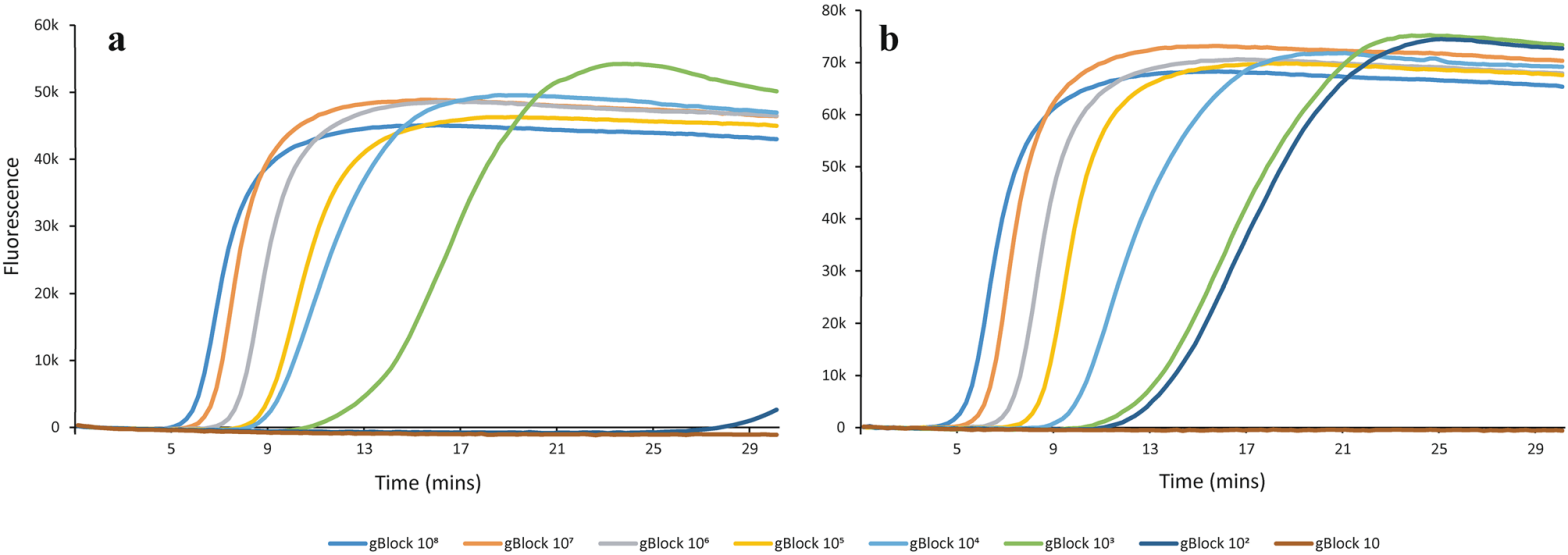
Detection limits of COI2377 (**a**) and CAD263 (**b**) LAMP assays evaluated using gBlock synthetic gene fragments with serial dilutions from 1 × 10^8^ copies/µL to 1 × 10^1^ copies/µL.

**Figure 4:**
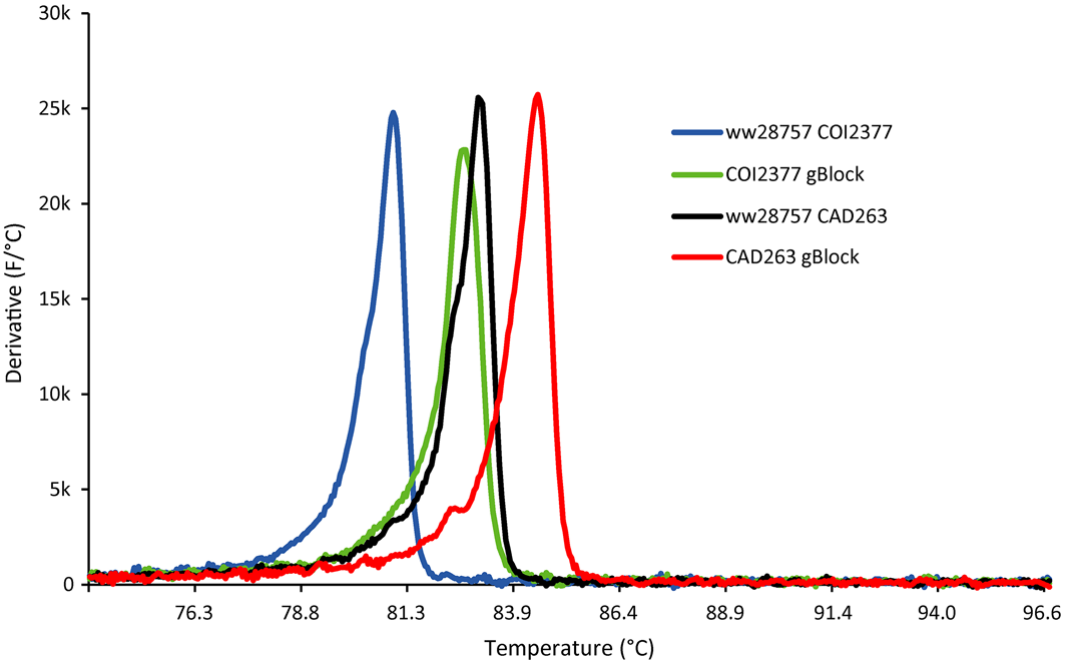
Comparison of the anneal derivations of gBlock positive control and *Liriomyza huidobrensis* DNA (sample ww28757) in the COI2377 and CAD263 LAMP assays. The COI2377 and CAD263 gBlock positives annealed at 82.69 and 84.40 °C, respectively. By contrast, for *L. huidobrensis* (sample ww28757), COI2377 and CAD263 LAMP products annealed at 80.98 and 83.00 °C, respectively.

Sensitivities of the COI2377 and CAD263 LAMP assays were determined using synthetic gBlock DNAs. The COI2377 assay was able to detect a minimum of 1000 copies/µL of DNA, with an anneal derivative of 82.6 ± 0.07 °C (Figs. 3a and 4). The CAD263 assay was slightly more sensitive with the detection limit at 100 copies/µL of DNA (Fig. 3b). The anneal derivative of CAD263 gBlock DNA was 84.1 ± 0.14 °C (Fig. 4). Anneal derivatives of the gBlocks were 1–2 °C higher than that observed among SLM positive samples (Fig. 4). These gBlocks were used as positive controls with the concentration of 1X 10^6^ copies/µL.

### Performance of the LAMP assays

Both COI2377 and CAD263 LAMP assays positively amplified all 184 SLM specimens within 25 min. Normally, positive signals can be detected within 10 min. When run on Genie III, the anneal derivative of COI2377 on SLM is 81.1 ± 0.13 °C, while CAD263 assays had an anneal derivative of 83.0 ± 0.01 °C. These assays, when conducted on a qPCR machine such as MIC, had a 1–2 °C higher melting temperature compared to the annealing temperature on GenieIII in both gBlock and samples. Generally, the positive signals were detected within 15 cycles (6.25 min) and 20 cycles (8.33 min) for COI2377 and CAD263 assays, respectively. Our LAMP assays were both highly specific to SLM with no amplification from any of the 146 specimens of the 15 non-target species (Table 2 and Supplementary Table S1).

**Table 2:** Leafminer species tested for both COI2377 and CAD263 LAMP assays

Species	Family	Samples	Collected from Hosts	State
*Liriomyza huidobrensis* (Blanchard, 1926)	Agromyzidae	184	*Amaranthus* sp., *Phaseolus vulgaris*, *Hibiscus trionum*, *Brassica* sp., *Capsicum annuum*, *Apium graveolens*, *Stellaria media*, *Cucumis sativus*, Asteraceae sp., *Vicia faba*, *Lactuca sativa*, *Beta vulgaris*, *Sonchus* sp., *Spinacia oleracea*, *Solanum lycopersicum*, *Trifolium repens*, *Brassica rapa* , *Cucurbita pepo*	NSW, QLD
*Liriomyza brassicae* (Riley, 1885)	Agromyzidae	13	*Brassica* sp., *Brassica juncea*, *Sonchus* sp.	NSW, QLD
*Liriomyza chenopodii* (Watt, 1924)	Agromyzidae	8	*Spinacia oleracea*	NSW
*Liriomyza sativae* (Blanchard, 1938)	Agromyzidae	5	*Macroptilium atropurpureum*	QLD
*Liriomyza trifolii* (Burgess, 1880)	Agromyzidae	26	*Helianthus annuus* and laboratory colony	NT, WA
*Liriomyza* sp.	Agromyzidae	1	*Chenopodium album*	QLD
*Calycomyza lantanae* (Frick, 1956)	Agromyzidae	4	*Lantana camara*	QLD
*Calycomyza humeralis* (Roser, 1840)	Agromyzidae	6	*Rumex crispus*, *Erigeron* sp.	QLD
*Chromatomyia syngenesiae* (Hardy 1849)	Agromyzidae	46	*Glebionis coronaria*, *Leucanthemum sp*, *Sonchus* sp.	NSW, QLD
*Phytomyza plantaginis* (Goureau, 1851)	Agromyzidae	3	*Plantago major*	NSW
*Scaptomyza australis* (Malloch, 1923)	Drosophilidae	13	*Brassica* sp., *Brassica juncea and Spinacia oleracea*	QLD, NSW
*Scaptomyza flava* (Fallen, 1823)	Drosophilidae	1	*Sonchus* sp.	NSW
*Tropicomyia polyphyta* (Kleinschmidt, 1961)	Agromyzidae	4	*Lilium lancifolium*, *Araujia sericifera*	QLD
sp. indet. #01	Chloropidae	1	*Brassica juncea*	QLD
sp. indet. #02	Agromyzidae	4	*Trifolium* sp.	QLD
sp. indet. #03	Chloropidae	11	*Spinacia oleracea*	NSW

Refer to Supplementary Table S1 for detailed specimen information.

**Figure 5:**
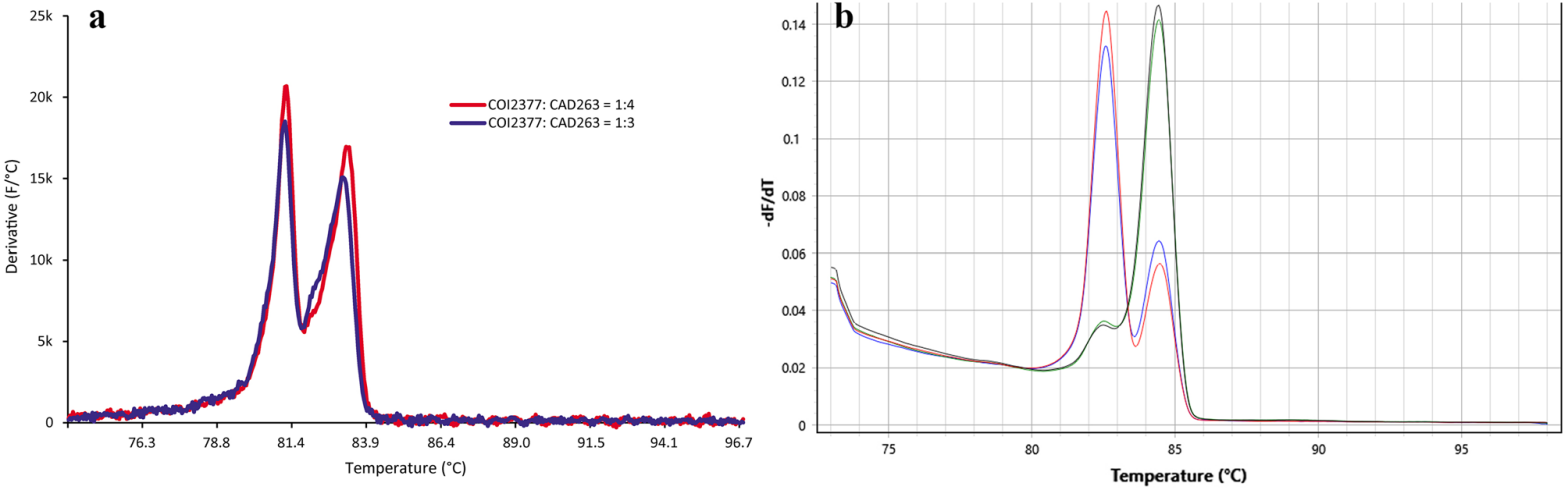
Anneal derivatives of multiplexed COI2377 and CAD263 LAMP assays. (**a**) single *Liriomyza huidobrensis* specimen assay exhibiting separate and similar intensity derivative peaks for COI2377 and CAD263 targets (primer master mix target ratios of 1:4 and 1:3; run on Genie III). (**b**) multiple *L. huidobrensis* specimen assays exhibiting separate but variable intensity derivative peaks for COI2377 and CAD263 targets (primer master mix ratio 1:3; run on MIC).

Multiplexes of the two LAMP assays initially tested on a single SLM specimen, consistently exhibited two distinct and equal intensity anneal peaks (Fig. 5a) when COI2377 and CAD263 primer ratios were set at 1:3 or 1:4 respectively. Multiplexes incorporating these primer ratios were inconsistently scored across 14 additional tested SLM specimens. In most replicates, the anneal peak of either of the two LAMP targets dominated the peak of the other (Fig. 5b), with no apparent trend to this biased amplification between the targets and no confident means to score if both targets had positively amplified.

### Naked eye monitoring with colorimetric indicator

**Figure 6:**
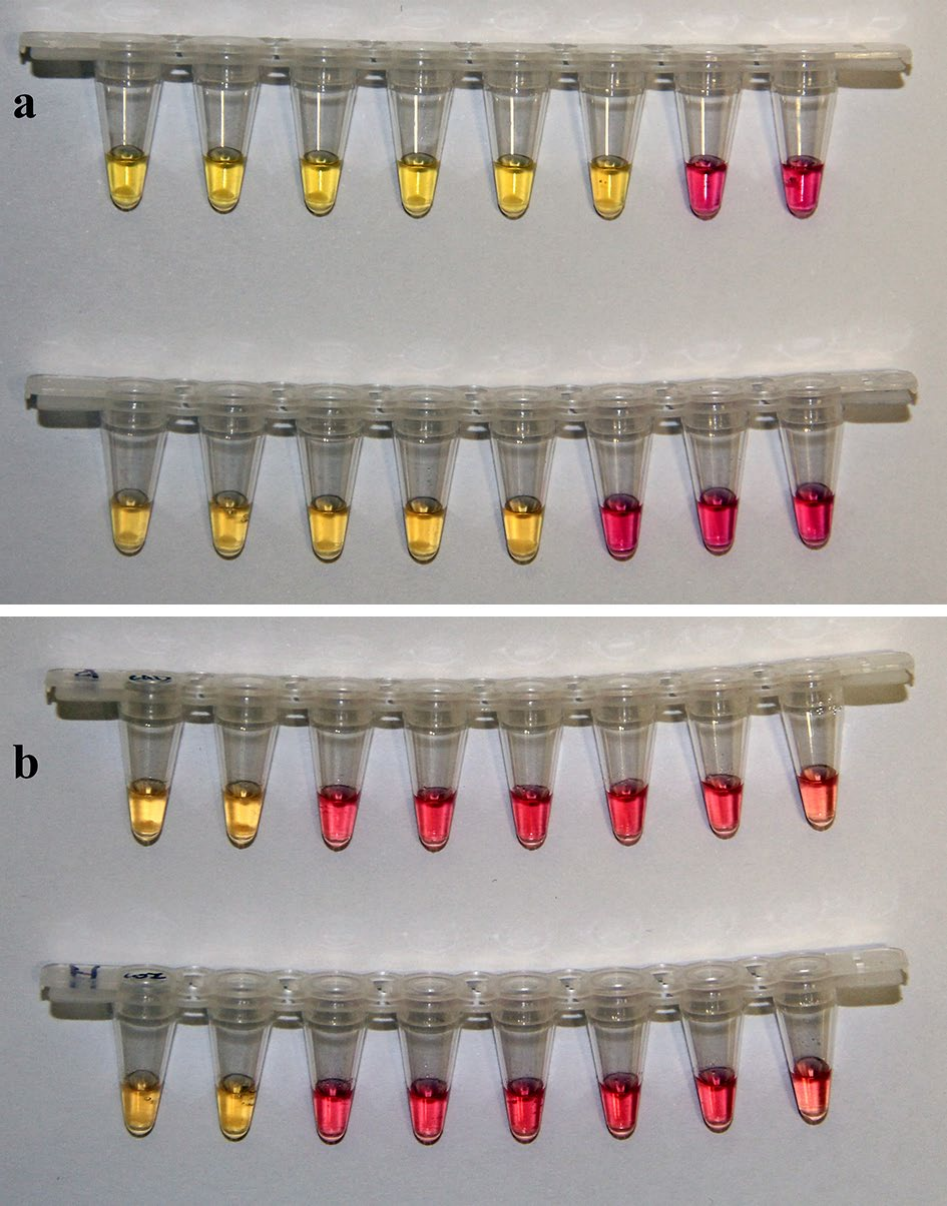
Sensitivity (**a**) and specificity (**b**) of the CAD263 and COI2377 LAMP assays using colorimetric mastermix. a) Sensitivity tests of the CAD263 (top) and COI2377 (below) LAMP assays on a 10 × dilution series from 10^8^ to 10^1^ copies/µL. (**b**) Specificity tests of the CAD263 (top) and COI2377 (below) LAMP assays. Samples tested from left to right were ww28757 *Liriomyza huidobrensis*, ww28758 *L. huidobrensis*, ww28727 *Scaptomyza australis*, ww28728 *Tropicomyia polyphyta*, ww28740 *Calycomyza lantanae*, ww28744 *L. brassicae*, ww28746 *L. trifolii* and no-template negative control. End-of-run positive reactions exhibited as a yellow colour change, negative reactions unchanged pink colour. Refer Supplementary Table S1 for detailed specimen information.

Crude isothermal heating of test reactions and use of a colorimetric LAMP MasterMix for equipment-free visualisation of LAMP positive products was successfully achieved after 30 min at single-plex COI2377 and CAD263 LAMP assays. Yellow stained positive LAMP reactions observed among all tested SLM specimens (N = 8) were readily discernible from the default pink stains observed in LAMP tests of negative controls and 12 non-target species (Fig. 6). Sensitivity of the colorimetric Master-Mix LAMP assays to gBlock targets was the same as that initially reported using the Optigene Isothermal Master mix (100 and1000 copies/µL at COI2377 and CAD263 respectively).

## Discussion

We developed two LAMP assays (COI2377 and CAD263) for specific genetic identification of SLM, under laboratory and in-field conditions. We tailored oligonucleotide primers in our LAMP assays to target short fragments of mitochondrial COI and nuclear CAD genes, each containing an array of fixed nucleotide positions that are unique to the species. Our *in-silico* comparisons of the primer suites against reported sequences at public repositories (BOLD and GenBank) indicated the primers were 100% compatible with SLM accessions of the target gene regions, and collectively unmatched to accessions of other reported Agromyzids and leafminers. We directly tested the specificity of our LAMP assays against 184 adult SLM sampled from affected sites in NSW, and QLD, and against 146 co-occurring specimens of 15 non-targeted leafminer species, including four other *Liriomyza* species. Positive LAMP detections were obtained exclusively from all tested SLM, and absent for all other tested taxa. We acknowledge that direct specificity testing of our LAMP primer sets was taxonomically limited mainly to species obtained recently at horticultural sites suspected to contain introduced leafminers. Subsequently our sample tested just a small portion of all possible leafminer species present in Australia. Further, many of the untested leafminer species in Australia also are unreported at public sequence repositories for the gene regions targeted by our LAMP assays. Consequently, our in-silico analyses also were taxonomically limited by availability of comparable taxa. This is a common issue affecting LAMP developments and validations. For target genera, such as *Liriomyza*, often sequence and specimen replicates are readily available for the focal pest species, but limited for other described taxa that are either rarely encountered or of restricted geographic distribution. Despite these shortcomings, our LAMP assays failed to amplify from other recently introduced *Liriomyza* pests in Australia (*L. sativae* and *L.trifollii*), common native *Liriomyza* (eg., *L. brassicae* and *L. chenopodii*) and other common leafminer taxa (eg., *Chromatomyia syngenesiae* and *Phytomyza plantaginis*). Both LAMP assays showed high SLM species specificity and sensitivity, and both can be used independently for rapid identification in Australia.

We designed two synthetic gBlock DNA fragments to accompany our LAMP assays. These gBlocks should be used as known quantity positive controls and are especially useful when annealing or melt curve analyses are performed following LAMP amplification. Compared to SLM LAMP products, amplicons of these gBlocks have a higher melting temperatures. Subsequently, suspected cross-contamination of test samples by gBlock positives can be readily detected. In addition, because gBlocks are synthesized short fragment DNAs, they are more stable than extracted specimen DNA. Therefore it is more reliable as a test templates for detection of false negatives resulting from degradation of LAMP primer/master mix stocks. We recommend using gBlock at a concentration of 10^6^ or higher, to match the fluorescent intensity generally observed from fresh SLM specimens.

Our LAMP assays are reliable for diagnostic detection of SLM under laboratory conditions using standard fluorometric thermal cyclers, and also potentially under in-field conditions using crude heating equipment for isothermal heating and colorimetric staining for simple visual observation of test results. In contrast to that reported by Zhang, et al.^32^, our colorimetric method did not reduce the sensitivity of our LAMP assays. However, it should be noted that this method does not allow annealing or melt curve analyses, which means contamination from the gBlocks or potential non-specific amplification could not be distinguished from a true positive.

As the COI2377 and CAD263 LAMP assays have different observable anneal derivative temperatures, we attempted to multiplex them as a simultaneous real-time PCR assay for use with q-PCR equipment. However, in most cases, either one of the assays dominated the reaction and the other only appeared as a shoulder peak (Fig. 5b). This may be due to the available ratios of mitochondrial and nuclear DNA extracted from among individuals, or to other efficiencies inherent in the amplification of the targeted gene fragments. Regardless, this is not of high concern as both assays can be used independently for simplified genetic identification of SLM.

In conclusion, we have developed two genetically independent LAMP assays that are specific and reliable for rapid genetic identification of SLM. Both assays were validated with adult and pupal specimens, and we expect the assays will work equally well on the larval specimens. Paired with fast and simple DNA extraction protocols (i.e., Xtract) these assays can be performed in-field within an hour and without need of expensive equipment. This will significantly accelerate the ready use of this diagnostic tool where there is need for rapid confirmation of a suspected presence of the pest. Both assays exhibited similar sensitivity and amplification time, offering users the flexibility to choose either one for diagnosing suspected SLM. In addition, the combination of the COI2377 and CAD263 assays could be used to investigate potential interspecific hybridization of *L. huidobrensis* and other species. Early in-field diagnostics facilitated by our LAMP assays will allow faster management responses to incursions and movement of this pest species.

## Methods

### Sampling

Ethanol (> 90%) preserved adult or pupal leafminer fly specimens were provided to us by various agencies (see acknowledgements) involved with SLM surveillances in NSW, NT, QLD and WA during 2020–2023. The specimens were either captured as adults or raised as adults emerging from larvae/pupae sampled from leaf-mined host plants.

Following all non-destructive DNA analyses, morphologically identified specimens were accessioned for curation at the Biosecurity Collections unit at Orange Agricultural Institute (NSW Dept. of Primary Industries). Retrospective DNA barcode identification of all specimens was conducted at the Wagga Wagga Agricultural Institute using protocols reported in Supplementary Methods S1. We deposited details of specimen sample records (Supplementary Table S1), their DNA barcodes and other associated sequences as a dataset (DS-SLMWW) “SLM and leafminers Australia”, released at the Barcode of Life Data (BOLD) systems repository (http://www.boldsystems.org/).

### LAMP designs and laboratory preparations

We targeted the mitochondrial cytochrome* c* oxidase subunit I (COI) gene as a primary DNA barcode locus for LAMP assay development based on its reported utility for molecular identification of Agromyzid species^33^. We used the extensive library of Agromyzid COI sequence accessions at BOLD and GenBank in that development. Additionally, this provided us with a means to test species identities of our sequenced specimens against taxonomically associated sequence accessions reported at the two repositories.

In addition to COI sequences, we obtained CAD gene sequences (partial) of 31 Agromyzids reported at GenBank and used these as an additional targeted gene sequence alignment for Agromyzid species identification and SLM LAMP development. This single-copy nuclear encoded gene has been reported for genetic identification of species in *Liriomyza*^28^ and other important Dipteran genera (eg., *Culicoides*)^34^. For this purpose it serves as an independent and bi-parentally inherited locus for comparative species analysis against the strictly maternally inherited mtDNA COI locus.

For the design of SLM-specific LAMP primers, we examined COI and CAD alignments to identify sequence strings containing variable nucleotide sites among Agromyzid species and conserved sites among SLM specimens. We used our in-house sequence library of Agromyzid species and sequences obtained from GenBank and BOLD for alignment, and PrimerExplorer version 5 http://primerexplorer.jp/e/index.html to design candidate LAMP primer-sets specific to the COI and CAD sequence of SLM using the default setting. All primers were synthesised by Sigma Aldrich (Merck, USA) with HPLC purification.

### LAMP assay optimization

We prepared a primer master mix for each LAMP assay. The outer primers (F3 and B3), inner primers (FIP and BIP) and the loop primers (LF and LB) were mixed as per the following ratios 1:6:3, 1:8:4, 1:10:5 and 1:12:6. Each LAMP assay (total volume 25 µL) consisted of 14 µL of Isothermal Master Mix (DR001, OptiGene, UK), 10 µL of primer master mix at various concentrations and 1 µL of test template. We optimized the LAMP assays on a Genie III (OptiGene, UK) at a temperature of 65 °C for 30 min followed by an annealing curve analysis from 98 to 73 °C ramped at 0.05 °C/s. After the optimization of primer concentration, we compared two Isothermal Master Mixes DR001 and DR004 (OptiGene, UK) both of which incorporate a fluorescent dsDNA intercalating dye. We selected the optimum conditions based on time of amplification and fluorescent intensity.

We evaluated the sensitivity of the LAMP assays using two gBlock DNAs (Table 1, IDT, USA) in tenfold serial dilution from 10^8^ copies/µL to 10 copies/µL. The sensitivity test was performed on Genie III with the optimized primer concentration and the assay condition as mentioned above. We recreated graphs of all amplification and derivative curves in this study using the data output from Genie III machine.

### LAMP primer specificity

We tested the specificity of both LAMP assays against 330 specimens comprising 16 species (Table 2 and Supplementary Table S1), including 184 SLM. The taxonomic identification of most specimens (216 out of 330) was confirmed through their COI sequences. Of the remaining specimens 106 were morphologically identified *L. huidobrensis* and 8 from laboratory colony of *L. trifolii*. The LAMP assays were conducted on a Genie III using the optimized condition or on a MIC qPCR machine (Bio molecular system, Australia) for higher throughput. On MIC, the cycling condition was: 60 cycles of a single step cycle at 65 °C for 25 s, followed by melt curve analysis from 73 to 98 °C with ramping at 0.05 °C/s. The amplification time was calculated as 25 s × Cq value.

### LAMP multiplexing

We used the above-mentioned 25 µL reaction system with 10 µL of primer mix consisting of COI and CAD LAMP primers in different ratios which ranged from 1:9 to 1:1. Initially, we multiplexed LAMP with a single SLM specimen on a Genie III to determine an optimal primer master ratio of COI and CAD. The optimal ratio was tested against 14 additional specimens of SLM, on a MIC qPCR.

### LAMP colorimetric detection

We conducted COI and CAD LAMP assays using crude heating in a 65 °C water bath and colorimetric staining to simulate in-field LAMP testing without specialised equipment used for isothermal heating and post-run scoring. LAMP assays contained 12.5  µL of WarmStart® Colorimetric LAMP 2X Master Mix (New England Biolabs, Australia), 1.6 µM of the FIP and BIP primers, 0.2 µM of the F3 and B3 primers, 0.4 µM of the Loop primers, 1 µL of target DNA, diluted to 25 µL total volume. LAMP reactions were run in 200 µL sealed microtubes and floated in a 65 °C water bath for 30 min. Post-run reactions were visually scored for colours observed among known SLM positives, negative controls and non-target species. Sensitivity of these colorimetric tests to target SLM DNA was determined against gBlocks run for 30 min on a PCR machine (Eppendorf Mastercycler, Germany).

### Supplementary Information

Supplementary Information.

Supplementary Table S1.

## Data Availability

The sequence data and specimen details are available from GeneBank under accession number OR038431 - OR038697 and Barcode of Life Data systems as a dataset (DS-SLMWW).
