# Supplementary material for: Two independent LAMP assays for rapid identification of the serpentine leafminer, Liriomyza huidobrensis (Blanchard, 1926) (Diptera: Agromyzidae) in Australia
Source: Sci Rep. 2023 Dec 15;13:22286. doi: 10.1038/s41598-023-49472-9 (PMC10721864; doi:10.1038/s41598-023-49472-9)
Supplement: Supplementary file 1 — Supplementary Information. [file 41598_2023_49472_MOESM1_ESM.docx]

**Two independent LAMP Assays for rapid identification of the serpentine leafminer, *Liriomyza huidobrensis* (Blanchard 1926) (Diptera: Agromyzidae) in Australia**

**Authors**: Xiaocheng Zhu^1*^, David Gopurenko^1^, Joanne C. Holloway^1^, John Duff^2^, Mallik B. Malipatil^3^

^1^ NSW Department of Primary Industries, Wagga Wagga Agricultural Institute, Wagga Wagga, NSW 2650 Australia

^2^ Queensland Department of Agriculture and Fisheries, Warrego Highway, Gatton, QLD 4343

^3^ Agriculture Victoria Research and La Trobe University, 5 Ring Road, Bundoora VIC 3083, Australia

*Corresponding author: xiaocheng.zhu@dpi.nsw.gov.au

ORCID ID: XZ 0000-0003-4468-1090, DG 0000-0003-2886-1614,

**Supplementary Materials**

**Supplementary** **Methods S1. DNA barcoding methods**

Leafminer specimens used in DNA barcoding were registered at the Barcode of Life Data systems (BOLD; http://www.boldsystems.org/). Specimen sampling details and associated specimen DNA barcodes & other gene sequences are available as a downloadable dataset “SLM and leafminers Australia” (DS-SLMWW), released at BOLD. Sequences were submitted via BOLD to the National Centre for Biotechnology Information (NCBI) and released with GenBank accession numbers (refer Supp. Table S1 for BOLD specimen records and associated GenBank accession numbers).

Specimen DNA extraction was preceded by a non-destructive tissue digestion. Whole leafminer fly specimens preserved in ethanol (90-95%) were transferred from collection vials into fresh microtubes allotted with individual alpha-numeric sample identifiers (ID) and incubated for half hours at room temperature to remove residual ethanol. Dried specimens were individually digested at 56º C overnight in 250 µL of DXT tissue digestion buffer (QIAGEN, Doncaster, Australia) incorporating 1% Proteinase K additive (QIAGEN). Following digestion, specimens were removed to new tubes containing 1mL of 90-95% ethanol for specimen curation at Biosecurity Collections Unit, Orange Agricultural Institute (NSW Dept. of Primary Industries), NSW Australia

DNA was extracted from 240 µL of each specimen digest using a KingFisher Flex robot and associated MagMAX CORE Nucleic Acid purification kits (Applied Biosystems). In some instances, speciemens were extracted manually using DNeasy Blood and tissue Kit (Qiagen). Final DNA eluted to 120 µL and were stored at -20° C.

Polymerase chain reactions (PCR) were prepared to a final volume of 15 µL using a MYRA Robotic Liquid Handling System (Bio Molecular Systems, Australia). PCR for DNA barcoding included 4 µL of DNA extract from single specimens, in the presence of Thermo Fischer Scientific reagents: 1X buffer, 2.8 mM MgCl2, 0.4 units of Platinum® Taq polymerase (Invitrogen), 200 μM dNTPs, and including 2 μM each of forward and reverse oligo-nucleotide primers (primers & primer combinations reported in Supplementary Table S2). Thermal cycling was completed using an Eppendorf Mastercycler ep gradient S PCR machine set with a universal thermal profile for all primer combinations: 95º C for 2 min; 40 cycles of 94º C for 30 s, 50º C for 30 s, 72º C for 45 s; 72º C for 5 min; storage at 4º C. PCR products stained with SYBR^TM^ Safe (Invitrogen) were visualized on a BioRad UV transilluminator after electrophoresis through a 1.5% agarose gel in 1% TAE buffer. Stained PCR products were qualitatively checked for expected fragment size against E-Gel 100bp ladder size marker (Invitrogen). PCR products sent to the Australian Genome Research Facility (Brisbane) were purified and bidirectional Sanger sequenced through an Applied Biosystems DNA Analyzer. Bidirectional sequence chromatograms were quality checked and assembled to sample ID using Lasergene SeqMan Pro ver. 8.1.0(3) (DNASTAR Inc., Maddison, WI, USA). Primer truncated sequences were aligned using BioEdit ver. 7.0.9.0^35^. All sequences were queried for species identity against publicly released sequence accession, using online engines: “Specimen Identification System” and “BLAST” respectively available at BOLD and GenBank. BOLD-allocated Barcode Index number (BIN) tagged to terminal clusters of similar DNA barcodes were auto-allocated at BOLD, to all compliant 5’ COI DNA barcodes^36^.

**Supplementary Table S2.**

Oligo-nucleotide primers and anneal direction in PCR amplification of Leafminer mitochondrial COI 5’, COI 3’ and Nuclear CAD gene region sequences. Primers include 5’ forward or reverse M13 sequence tails (upper case) and gene specific sequence (lower case). Source references of primers as indicated. M13 tails reported here are as currently used at AGRF, differ marginally from tails reported in several of the source references.

| Target | primer | Direction | Sequence (5′ – 3′) | References |
| --- | --- | --- | --- | --- |
| *COI 5’* | *BC1Fm* | F | TGTAAAACGACGGCCAGTtcwacwaaycayaargayatygg | Folmer, et al. ^37^ |
| *COI 3’* | *SCOI C1-J-2183-fm* | F | TGTAAAACGACGGCCAGTcaacatttatttgattttttgg | Simon, et al. ^38^ |
| *COI 5’* | *LR1m* | F | CAGGAAACAGCTATGACCtaaacttctggatgtccaaaaaatca | Hebert, et al. ^39^ |
| *COI 3’* | *SCOI TL2-N-3014-rm* | R | CAGGAAACAGCTATGACCtccaatgcactaatctgccatatta | Simon, et al. ^38^ |
| *CAD* | Lir-CAD-53Fm | F | TGTAAAACGACGGCCAGTatgagaaagatgaatatggyatgcc | Carapelli, et al. ^40^ |
| *CAD* | Lir-CAD-689Rm | R | CAGGAAACAGCTATGACCtgrccrcgattaccaaatttcat | Carapelli, et al. ^40^ |

**References**

35 Hall, T. A. BioEdit: a user-friendly biological sequence alignment editor and analysis program for Windows 95/98/NT. *Nucleic Acids Symposium Series* **41**, 95-98 (1999).

36 Ratnasingham, S. & Hebert, P. D. A DNA-based registry for all animal species: the barcode index number (BIN) system. *PLoS One* **8**, e66213 (2013).

37 Folmer, O., Black, M., Hoeh, W., Lutz, R. & Vrijenhoek, R. DNA primers for amplification of mitochondrial cytochrome c oxidase subunit I from diverse metazoan invertebrates. *Mol. Mar. Biol. Biotechnol.* **3**, 294-299 (1994).

38 Simon, C. *et al.* Evolution, Weighting, and Phylogenetic Utility of Mitochondrial Gene Sequences and a Compilation of Conserved Polymerase Chain Reaction Primers. *Ann. Entomol. Soc. Am.* **87**, 651-701 (1994).

39 Hebert, P. D. N., Penton, E. H., Burns, J. M., Janzen, D. H. & Hallwachs, W. Ten species in one: DNA barcoding reveals cryptic species in the neotropical skipper butterfly *Astraptes fulgerator*. *Proceedings of the National Academy of Sciences of the United States of America* **101**, 14812-14817 (2004).

40 Carapelli, A. *et al.* Cryptic Diversity Hidden within the Leafminer Genus *Liriomyza* (Diptera: Agromyzidae). *Genes (Basel)* **9** (2018).
